# Data classification of magnetic resonance tomography and computer tomography images of brain in parturients with neurological complications of eclampsia

**DOI:** 10.1186/cc10924

**Published:** 2012-03-20

**Authors:** G Tikhova, E Shifman

**Affiliations:** 1 Kulakov Scientific Center of Obstetrics, Gynecology and Perinatology, Moscow, Russia

## Introduction

The goal of the study was to classify protocol data recorded during magnetic resonance tomography (MRT) and computer tomography (CT) examinations of the brain in patients with neurological complications of eclampsia; to define the MRT/CT examination data structure; and to perform frequency analysis of main MRT/CT characteristics and estimate their frequency distributions defined by studied pathology. The data included in the study were reported in medical journals and met definite criteria for inclusion.

## Methods

We collected cases of neurological complications of eclampsia reported in English-language medical journals from 1980 to 2008. The study methods include structural and frequency analysis of brain MRT/CT image protocols.

## Results

The analyzed sample included 77 cases of neurological complications of eclampsia. We extracted the following positions from the plain texts of MRT/CT descriptions: brain injury areas (occipital, temporal, parietal and frontal lobes); injury depth (cortical and/or subcortical matter); brain structures undergoing injury (classification was too complicated); injury nature (vasogenic/ischemic edema, hemorrhage). Abnormalities in occipital (84.6%) and parietal (70.7%) lobes were the most frequent, injuries in temporal lobes were quite rare (26.9%), but damage in frontal lobes was the most uncommon (24.4%). Combined injury in occipital and parietal lobes was recorded in more than two-thirds of cases (72.4%). Combined injury in occipital-frontal lobes (29.3%) and occipital-temporal (27.6%) lobes were observed in almost one-third of patients. Synchronous injury in the temporal and frontal lobes was the least common (6.9%). Simultaneous damage of three and more lobes was observed quite rarely (14.6%). Most abnormalities were bilateral with frequency not less than 78.0%. Unsymmetrical injury observed in some patients was located in the right lobe in most cases. All analyzed cases include only 7.1% of single left injury and all of them were located in the occipital lobe. Vasogenic edema occurred in 83.5% of cases, while ischemic damage was observed in 10.4%. The incidence of hemorrhage was 6.1%.

## Conclusion

The analysis reveals a general picture of the most distinctive features of brain damage following neurological complications of eclampsia.

